# Burden of illness in patients with pulmonary hypertension due to interstitial lung disease: a real-world analysis

**DOI:** 10.1186/s12890-024-03141-3

**Published:** 2024-07-11

**Authors:** Gustavo Heresi, Bonnie Dean, Benjamin Wu, Henry Lee, Margaret R. Sketch, Dana Stafkey-Mailey, Kellie Morland, Peter Classi, Leslie Spikes

**Affiliations:** 1 Department of Pulmonary and Critical Care Medicine, Respiratory Institute, Cleveland Clinic, 9500 Euclid Avenue A90, 22195 Cleveland, Bonnie Dean, OH USA; 2 Cencora, Inc, 1 West First Ave, 55 T.W. Alexander Drive, Conshohocken, PA USA; 3 United Therapeutics Corporation, 55 T.W. Alexander Drive, 27709 Durham, NC USA; 4 University of Kansas Medical Center, 3901 Rainbow Boulevard, Kansas City, KS 66160 USA

**Keywords:** Pulmonary hypertension, Interstitial lung disease, Cost, Database analysis, Healthcare resource utilization, Medical claims, Treatment patterns

## Abstract

**Background:**

Pulmonary hypertension due to interstitial lung disease (PH-ILD) is associated with high rates of respiratory failure and death. Healthcare resource utilization (HCRU) and cost data are needed to characterize PH-ILD disease burden.

**Methods:**

A retrospective cohort analysis of the Truven Health MarketScan^®^ Commercial Claims and Encounters Database and Medicare Supplemental Database between June 2015 to June 2019 was conducted. Patients with ILD were identified and indexed based on their first claim with a PH diagnosis. Patients were required to be 18 years of age on the index date and continuously enrolled for 12-months pre- and post-index. Patients were excluded for having a PH diagnosis prior to ILD diagnosis or the presence of other non-ILD, PH-associated conditions. Treatment patterns, HCRU, and healthcare costs were compared between the 12 months pre- versus 12 months post-index date.

**Results:**

In total, 122 patients with PH-ILD were included (mean [SD] age, 63.7 [16.6] years; female, 64.8%). The same medication classes were most frequently used both pre- and post-index (corticosteroids: pre-index 43.4%, post-index 53.5%; calcium channel blockers: 25.4%, 36.9%; oxygen: 12.3%, 25.4%). All-cause hospitalizations increased 2-fold, with 29.5% of patients hospitalized pre-index vs. 59.0% post-index (*P* < 0.0001). Intensive care unit (ICU) utilization increased from 6.6 to 17.2% (*P* = 0.0433). Mean inpatient visits increased from 0.5 (SD, 0.9) to 1.1 (1.3) (*P* < 0.0001); length of stay (days) increased from 5.4 (5.9) to 7.5 (11.6) (*P* < 0.0001); bed days from 2.5 (6.6) to 8.0 (16.3) (*P* < 0.0001); ICU days from 3.8 (2.3) to 7.0 (13.2) (*P* = 0.0362); and outpatient visits from 24.5 (16.8) to 32.9 (21.8) (*P* < 0.0001). Mean (SD) total all-cause healthcare costs increased from $43,201 ($98,604) pre-index to $108,387 ($190,673) post-index (*P* < 0.0001); this was largely driven by hospitalizations (which increased from a mean [SD] of $13,133 [$28,752] to $63,218 [$75,639] [*P* < 0.0001]) and outpatient costs ($16,150 [$75,639] to $25,604 [$93,964] [*P* < 0.0001]).

**Conclusion:**

PH-ILD contributes to a high HCRU and cost burden. Timely identification, management, and treatment are needed to mitigate the clinical and economic consequences of PH-ILD development and progression.

## Background

Pulmonary hypertension (PH) is a chronic condition characterized by elevated resting pulmonary arterial pressure with accompanying increased pulmonary vascular resistance on right heart catheterization (RHC) [1, 2] PH can be categorized into 5 groups, with Group 3 PH arising from underlying lung diseases and/or hypoxia, including interstitial lung disease (ILD) [1, 2]. ILD is heterogeneous, representing more than 200 disorders, and typically causes fibrosis and inflammation within the interstitial space (i.e., the tissue surrounding the lung’s air sacs, blood vessels, and airways) [3]. The pathologic changes of PH-ILD impair gas exchange and lead to breathlessness, an increased need for supplemental oxygen, reduced exercise and functional capacity, and diminished quality of life. Patients with PH-ILD and progressive pulmonary fibrosis can decline rapidly and have particularly high rates of respiratory failure and death [1, 3, 4].

PH-ILD prevalence varies widely due to variations in diagnostic techniques, the hemodynamic thresholds applied to define PH, the underlying ILD type, and disease stage [5]. PH has been reported in increasingly higher proportions of patients with idiopathic pulmonary fibrosis (IPF; the most commonly researched form of ILD) in a manner that is dependent on the timing of evaluation. Epidemiologic data indicate that PH prevalence is 8–15% at initial IPF diagnosis, 29–46% at the time of evaluation for lung transplant [4, 6–10] and up to 86% at the time of lung transplant [1, 4, 11–13]. Patients with PH-IPF have significantly worse survival than patients with IPF alone. In one analysis of patients with IPF undergoing RHC, median survival from the time of initial evaluation was 20.8 months for patients with PH (defined as mean pulmonary artery pressure [mPAP] > 20 mmHg) versus 37.5 months for patients without PH (*P* = 0.001) [11].

Patients with fibrotic ILDs can benefit from supportive care, supplemental oxygen, and pulmonary rehabilitation, especially early in the course of disease [3, 14]. Pharmacotherapeutic treatments for PH-ILD vary based on the underlying etiology, with evidence-based drug treatments existing for only a small number of ILDs [3]. Off-label drug use is common and includes anticoagulants, corticosteroids, immunomodulators, and pulmonary vasodilators, although the clinical impact of these drugs is disputed [3, 14]. Two antifibrotic therapies have been approved for patients with IPF (nintedanib and pirfenidone), but their effect on PH attributed to IPF has not been studied [5].

Although medications approved for pulmonary arterial hypertension (PAH) have been studied in patients with PH-ILD, efficacy has not been shown [1]. Research evaluating the endothelin receptor antagonists, (ERAs) ambrisentan, bosentan, and macitentan, failed to show improvements in 6-minute walk distance (6MWD), forced vital capacity, symptoms, hemodynamics, or time to clinical worsening or death in PH-ILD [1, 15–20]. Likewise, patients with PH-ILD treated with the phosphodiesterase-5 inhibitor (PDE5i), sildenafil, showed no benefit in 6MWD or perceived exertion, although one study found improved oxygen saturation and patient quality of life [1, 21, 22]. Last, a study evaluating the soluble guanylate cyclase stimulator (sGCS), riociguat, in patients with PH-ILD was terminated early due to patient harm [1, 23].

Until recently, no medical therapies were approved for PH-ILD by the United States Food and Drug Administration (FDA) [5]. However, in 2021, inhaled treprostinil received an FDA indication to improve exercise ability in patients with Group 3 PH-ILD [24, 25]. This approval was based on results from the phase 2/3, randomized, double-blind, placebo-controlled INCREASE trial, which successfully achieved the primary endpoint of change in 6MWD from baseline to week 16 [26].

Fibrotic ILDs are associated with high healthcare resource utilization (HCRU) and costs, especially in patients with more advanced disease [27]. However, to date, only one US study (a retrospective analysis, conducted from 2010 to 2013) has quantified treatment patterns, HCRU, and healthcare-related costs for patients with Group 3 PH (including patients with PH-ILD) [28]. More current and diagnostically specific real-world data are needed. This retrospective US medical claims analysis was conducted to characterize pharmacologic treatment patterns, HCRU, and costs in patients, both before and after PH diagnosis in patients with ILD.

## Methods

### Study design and data source

Adults with a diagnosis of PH secondary to ILD were identified from the Truven Health MarketScan^®^ Commercial Claims and Encounters Database (CCAE) and Medicare Supplemental Database (now the Merative™ MarketScan^®^ Research Commercial and Medicare databases). The CCAE Database included annualized inpatient, outpatient, and pharmaceutical claims for nearly 51 million enrollees in > 150 commercial health plans located in the 50 states, and US territories. The Medicare Supplemental Database contained inpatient and outpatient medical and prescription claims for Medicare-eligible individuals with supplemental insurance offered by their former employers; approximately 4.3 million persons are enrolled annually in this database. All data used for this study were accessed in compliance with the conditions set forth in Sect. 164.514(a)-(b)(1)ii of the Health Insurance Portability and Accountability Act of 1996 Privacy Rule. All databases used are statistically certified as de-identified and no electronic or paper copies of medical charts were available. Therefore, informed consent and Institutional Review Board approval were not required.

### Study population

**Fig. 1:**
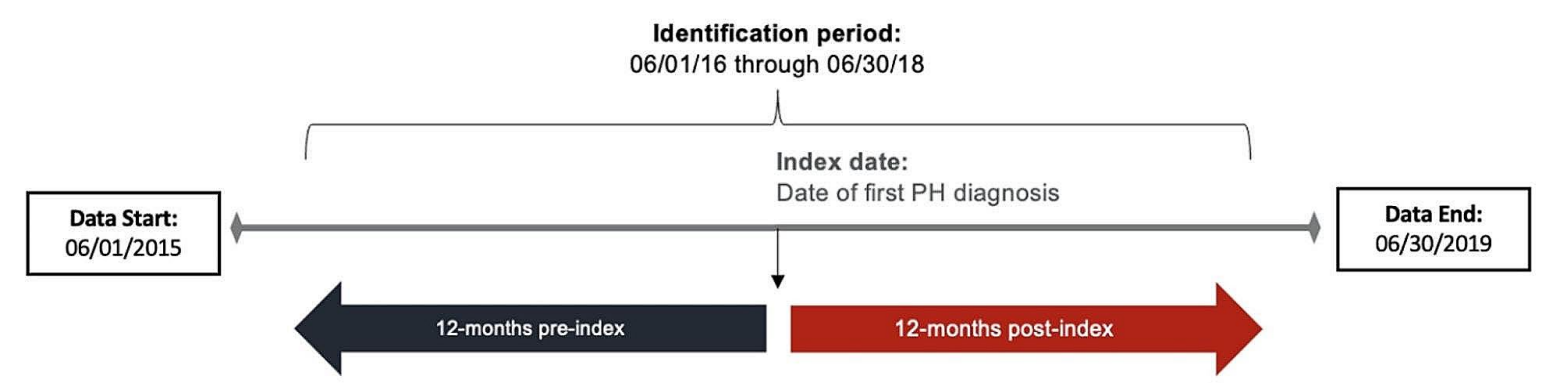
Study Design

Patients were required to have ≥ 1 medical claim with an ILD diagnosis between June 2015 and June 2018, and ≥ 1 subsequent medical claim for PH from June 2016 to June 2018 during the identification period (Fig. 1). The first observed claim with a PH diagnosis during the identification period was defined as the index date. At index, patients had to be ≥ 18 years of age, with ≥ 12 months of continuous pre- and post-index health insurance enrollment. Also required was ≥ 1 medical claim with an ILD diagnosis in the 12-month pre-index period. Patients were excluded if they had ≥ 1 medical claim for PH prior to ILD diagnosis, or if they had ≥ 1 medical claim diagnosing a non-ILD Group 3 PH condition or a Group 2, 4, or 5 PH condition at any time during the study [29]. Patients were excluded if they had only 1 outpatient claim or only 2 outpatient claims < 30 days apart for PH during the identification period.

### Pre- and post-index measures

Baseline demographic and clinical characteristics were measured in the pre-index period and included age, sex, geographic region, insurance payer and plan type, index year, Quan-Charlson comorbidity index (Quan-CCI) and individual comorbidities [30], and RHC frequency. Treatments for antifibrotic agents (i.e., nintedanib and pirfenidone); anti-reflux medications (e.g., antacids, proton pump inhibitors); calcium channel blockers; corticosteroids; oxygen; and pharmaceuticals approved for PAH (i.e., prostacyclin analogues, ERAs, PDE5i, and sGCS) were evaluated in the 12-months pre- and post-index periods.

All-cause HCRU was captured during the 12-month pre-and post-index periods and included the number and percentage of patients with ≥ 1 inpatient hospitalization, intensive care unit (ICU) admission during a hospitalization, emergency department (ED) visit, physician office visit, laboratory visit, and other outpatient visit. The number of unique prescriptions per-patient was also captured, as were per-patient hospital bed days (the sum of length of stay [LOS] days for all hospitalizations during the study period) averaged across the entire population; per-patient average LOS per hospitalization (averaged across patients with ≥ 1 hospitalization); and per-patient ICU days, averaged across those with ≥ 1 ICU stay.

Information regarding the setting of care was identified from the claims data using inpatient files, place of service variables, and applicable Current Procedural Terminology (CPT^®^)-4 procedure codes. ED and physician office visits were not counted if the visit occurred on the same day as a hospitalization. Laboratory visits were identified using claims for primary procedure codes in claims not previously categorized as ED, office, or ambulatory surgery visits. “Other” outpatient visits included visits not previously classified as ambulatory surgery, ED, physician office, or laboratory visits. Also classified as “Other” outpatient visits were any inpatient visits captured in the outpatient data file, but not documented as an inpatient visit in the inpatient data file.

Healthcare costs included both medical and prescription costs, and reflected payments made by both the health plan and patient (copayments, coinsurance, deductibles) during the 12-month pre- and post-index periods. Costs were reported as an average per-patient and included total costs, prescriptions, and related to the setting of care (inpatient or outpatient). Cost components related to inpatient care were hospitalizations (including ICU stays); for outpatient care, costs were reported for ED, physician office, laboratory, and other outpatient visits.

Patients with evidence of capitated claims (defined as ≥ 1 medical claim with a capitated service claim indicator) during the study period were excluded from cost analyses because capitated payments are pre-calculated and may not reflect the actual costs of care [31]. All costs were adjusted to 2019 US dollars using the Bureau of Labor Statistics’ medical care component of the Consumer Price Index [32], and reported annually.

### Statistical analysis

All demographic, clinical characteristics, and pharmacotherapy variables measured during the pre-index period were analyzed descriptively using frequencies and percentages for categorical variables, and means, standard deviations (SDs), medians, and interquartile ranges (IQRs) for continuous variables. For treatment patterns, McNemar’s test was used to compare differences in the proportion of patients who used treatments between the pre- and post-index periods. HCRU was calculated annually and assessed both pre- and post-index as frequencies and percentages as well as means (SD) and medians (IQR) and then compared using Wilcoxon non-parametric tests. Post-index costs were calculated annually and compared with pre-index costs using Wilcoxon non-parametric tests. All statistical tests applied a 2-sided hypothesis of no difference between the pre- and post-index periods, at a significance level of 0.05. Analyses were conducted using Statistical Analysis Software (Version 9.4 or higher; Cary, NC: SAS Institute, Inc.; 2011).

## Results

### Demographic and clinical characteristics

**Fig. 2:**
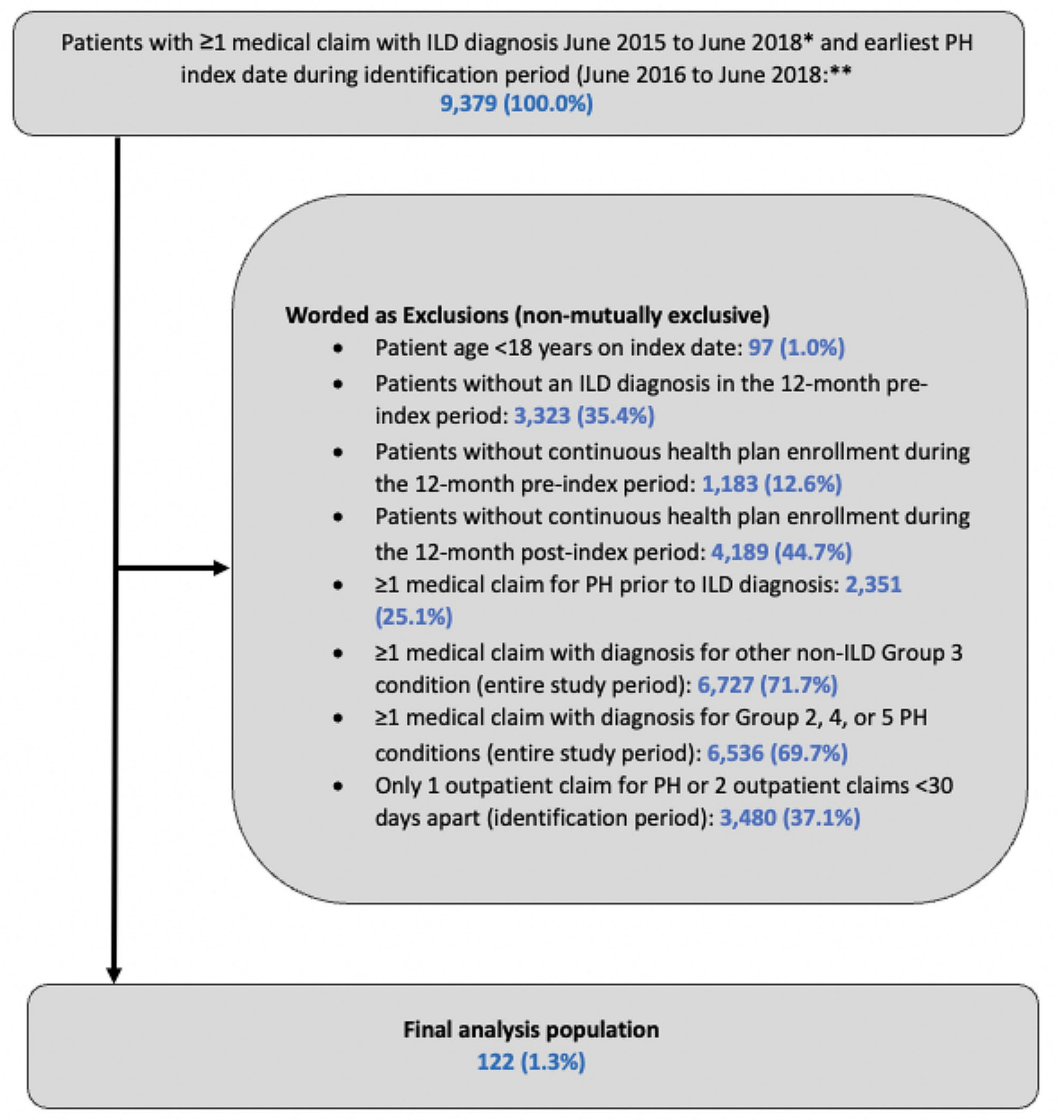
Patient Attrition. Abbreviations: ICD = International Classification of Disease; ILD = interstitial lung disease; PH = pulmonary hypertension. * Diagnostic codes, ICD-9-CM: 495.X, 500–505, 506, 506.4, 506.9, 508.1, 508.8, 515, 516.1, 516.2, 516.3X, 516.9, 517.1, 710.1, 710.3, 710.4, 714.81; ICD-10-CM: J17, J60.X-J67.X, J68, J68.4, J68.9, J70.1, J70.3, J70.4, J70.8, J82, J84.02, J84.04, J84.1X, J84.2, J84.89, J84.9, M05.1X, M32.13, M33.01, M33.11, M33.21, M33.91, M34.81. ** Diagnostic codes, ICD-9-CM: 416.0, 416.8, 416.9; ICD-10-CM: I27.0, I27.2, I27.20, I27.21, I27.23, I27.89, I27.9)

A total of 9,379 patients with a claim for ILD (June 2015 to June 2018) and a subsequent claim for PH (June 2016 to June 2018) were identified. After applying the selection criteria (Figs. 2), 122 patients with PH-ILD were included in the final cohort. Mean (SD) patient age was 63.7 (16.6) years, and most patients were female (64.8%) and had commercial insurance (54.9%). The highest proportion of patients resided in the geographic south (32.0%), and most (57.4%) had a Quan-CCI score ≥ 3 (Table 1). The most common prevalent comorbidities were chronic pulmonary disease (94.3%), diabetes (36.1%), and rheumatic disease (34.4%). In total, only 3.3% of patients had received RHC at baseline.

**Table 1:** Patient demographic and clinical characteristics at Index

	PH-ILD (*N* = 122)
**Age (years)**,** mean ± SD**	63.7 ± 16.6
18–34 years, n (%)	4 (3.3)
35–44 years, n (%)	13 (10.7)
45–54 years, n (%)	19 (15.6)
55–64 years, n (%)	32 (26.2)
65+	54 (44.3)
**Sex**, female, n (%)	79 (64.8)
**Region**,** n (%)**	
Northeast	30 (24.6)
North Central	31 (25.4)
South	39 (32.0)
West	22 (18.0)
**Insurance**,** n (%)**	
Commercial	67 (54.9)
Medicare supplemental	55 (45.1)
**Index year**,** n (%)**	
2016	49 (40.2)
2017	53 (43.4)
2018	20 (16.4)
**Quan-CCI**,** mean ± SD**	3.2 ± 1.9
**Quan-CCI categories**,** n (%)**	
1	23 (18.9)
2	29 (23.8)
3+	70 (57.4)
**Quan-CCI comorbidities**,** n (%)**	
MI	9 (7.4)
CHF	31 (25.4)
PVD	34 (27.9)
CVD	12 (9.8)
Dementia	2 (1.6)
CPD	115 (94.3)
Rheumatic disease	42 (34.4)
Peptic ulcer disease	3 (2.5)
Mild liver disease	8 (6.6)
Diabetes	44 (36.1)
Hemiplegia/paraplegia	4 (3.3)
Renal disease	17 (13.9)
Malignancy (any)	12 (9.8)
Moderate or severe liver disease	3 (2.5)
**Procedures**,** n (%)**	
Right heart catheterization	4 (3.3)
Echocardiography	48 (39.3)
CT imaging	72 (59.0)
Pulmonary function test	53 (43.4)
NT-proBNP	17 (13.9)
Lung ventilation-perfusion (V/Q) scan	3 (2.5)
Lung biopsy	3 (2.5)
Pulmonary stress testing	4 (3.3)
Pulmonary rehabilitation	28 (23.0)

Abbreviations: Quan-CCI = Quan-Charlson comorbidity Index; CHF = congestive heart failure; CPD = chronic pulmonary disease; CT = computed tomography; CVD = cerebrovascular disease; ILD = interstitial lung disease; MI = myocardial infarction; PH = pulmonary hypertension; PVD = peripheral vascular disease; SD = standard deviation.

### Pharmaceutical treatment patterns

**Fig. 3:**
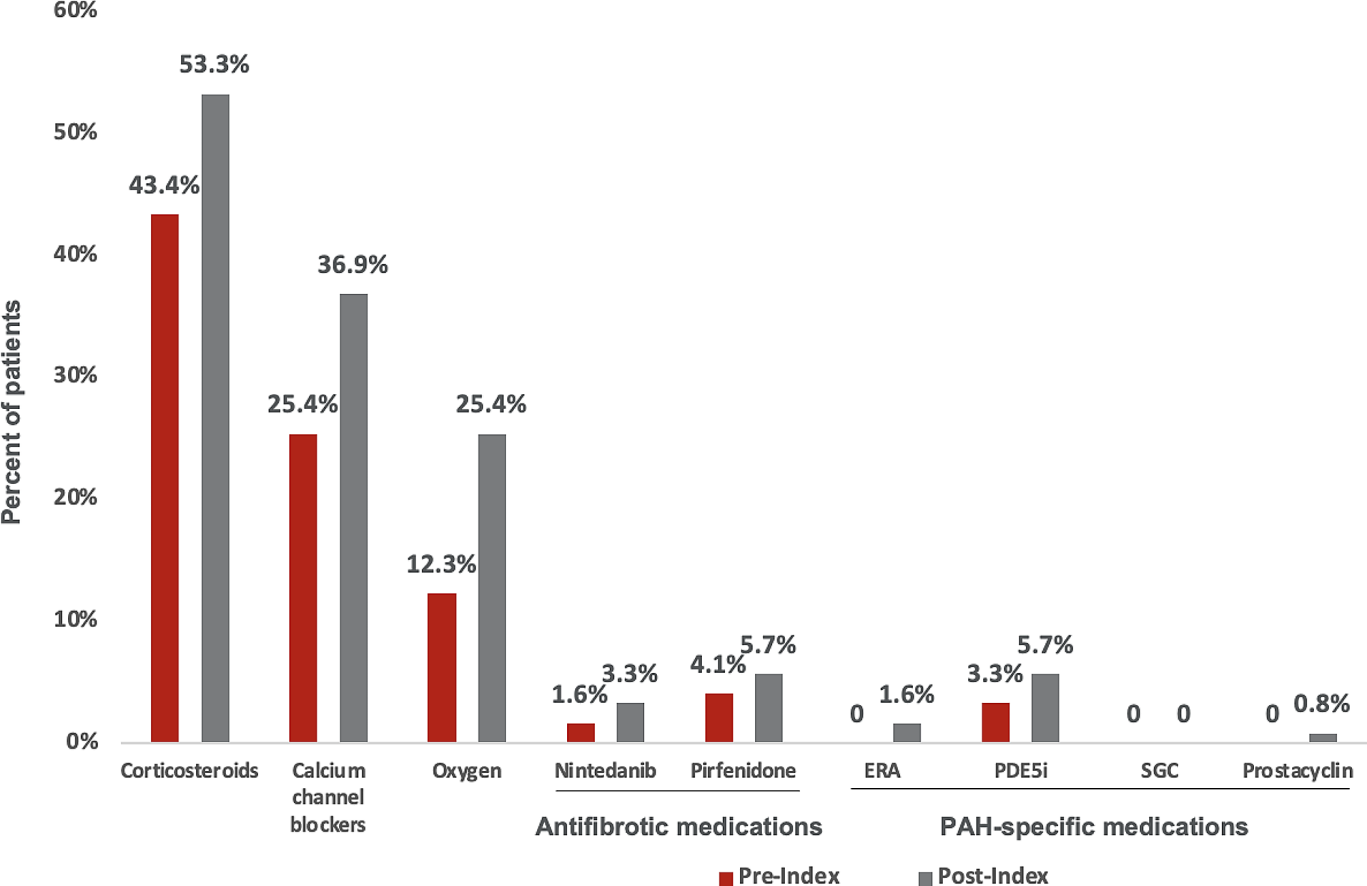
Treatment Regimens In the 12-Month Pre- and Post-Index Period. Abbreviations: ERA = endothelin receptor antagonist; PAH, pulmonary arterial hypertension; PDE5i = phosphodiesterase-5 inhibitor; SGC = soluble guanylate cyclase stimulator

The medication classes most commonly used prior to index continued to be those most commonly used post-index: corticosteroids (pre-index, 43.4%; post-index, 53.5%), calcium channel blockers (25.4%; 36.9%), and oxygen (12.3%; 25.4%) (Fig. 3). Of note, antifibrotic medications and PAH-specific medications were rarely used before or after the index date; PDE5i were the only PAH medication used in > 2% of patients (pre-index, 3.3%; post-index, 5.7%).

### Healthcare resource utilization and costs

Table 2 summarizes HCRU in terms of proportional and mean resource utilization in the 12 months pre- and post-index, including inpatient and outpatient care and unique prescriptions dispensed. Percentage of patients experiencing a hospitalization increased from 29.5% pre-index to 59.0% post-index (*P* < 0.0001), while ICU utilization increased from 6.6 to 17.2% (*P* = 0.0433). The mean number of inpatient visits, LOS per hospitalization, hospital bed days, and ICU days were all significantly higher post-index relative to pre-index. Between the pre-index and post-index periods, inpatient visits increased from a mean of 0.5 (SD, 0.9) to 1.1 (1.3) (*P* < 0.0001); LOS increased from 5.4 (5.9) to 7.5 (11.6) days (*P* < 0.0001); bed days increased from 2.5 (6.6) to 8.0 (16.3) (*P* < 0.0001); and ICU days increased from 3.8 (2.3) to 7.0 (13.2) (*P* = 0.0362).

**Table 2:** Healthcare Resource Utilization in the 12-Month pre- and Post-index Period

Healthcare Resources		Pre-index (*N* = 122)	Post-index (*N* = 122)	*P*-value
**Inpatient utilization**				
Inpatient visits*	N (%)	36 (29.5)	72 (59.0)	< 0.0001
	Mean (SD)	0.5 (0.9)	1.1 (1.3)	< 0.0001
	Median (IQR)	0 (0.0–1.0)	1.0 (0.0–2.0)	
Length of stay per hospitalization, days†	Mean (SD)	5.4 (5.9)	7.5 (11.6)	< 0.0001
	Median (IQR)	4.0 (2.0–7.0)	4.0 (3.0–8.0)	
Bed days*	Mean (SD)	2.5 (6.6)	8.0 (16.3)	< 0.0001
	Median (IQR)	0 (0.0–2.0)	2.0 (0.0–9.0)	
ICU days§	N (%)	8 (6.6)	21 (17.2)	0.0433
	Mean (SD)	3.8 (2.3)	7.0 (13.2)	0.0362
	Median (IQR)	3.5 (2.0–5.0)	3.0 (2.0–5.0)	
**Outpatient utilization**				
ED visits*	N (%)	46 (37.7)	42 (34.4)	0.6358
	Mean (SD)	0.6 (1.0)	0.5 (0.9)	0.6308
	Median (IQR)	0 (0.0–1.0)	0.0 (0.0–1.0)	
Physician office visits*	N (%)	114 (93.4)	115 (94.3)	1.0000
	Mean (SD)	14.0 (10.6)	15.9 (12.2)	0.2537
	Median (IQR)	13.0 (7.0–19.0)	13.0 (7.0–23.0)	
Laboratory visits*	N (%)	106 (86.9)	108 (88.5)	0.8318
	Mean (SD)	5.1 (4.6)	6.9 (8.1)	0.0560
	Median (IQR)	4.0 (1.0–8.0)	5.0 (2.0–8.0)	
Other outpatient visits*	N (%)	120 (98.4)	120 (98.4)	1.0000
	Mean (SD)	12.5 (13.3)	19.0 (17.7)	< 0.0001
	Median (IQR)	8.0 (4.0–16.0)	13.0 (6.0–23.0)	
Overall outpatient visits*	N (%)	121 (99.2)	121 (99.2)	1.0000
	Mean (SD)	24.5 (16.8)	32.9 (21.8)	< 0.0001
	Median (IQR)	21.5 (12.0–32.0)	28.0 (16.0–45.0)	
Unique prescriptions	Mean (SD)	37.5 (34.4)	46.4 (38.6)	< 0.0001
	Median (IQR)	30.0 (14.0–46.0)	38.0 (18.0–62.0)	

*Mean (SD) and median are calculated for the entire cohort

† Mean (SD) and median are calculated for the subset of patients with an inpatient hospitalization.

§ Mean (SD) and median are calculated for the subset of patients with an ICU stay during an inpatient hospitalization.

Abbreviations: ED = emergency department; ICU = intensive care unit; IQR = interquartile range; SD = standard deviation.

**Fig. 4:**
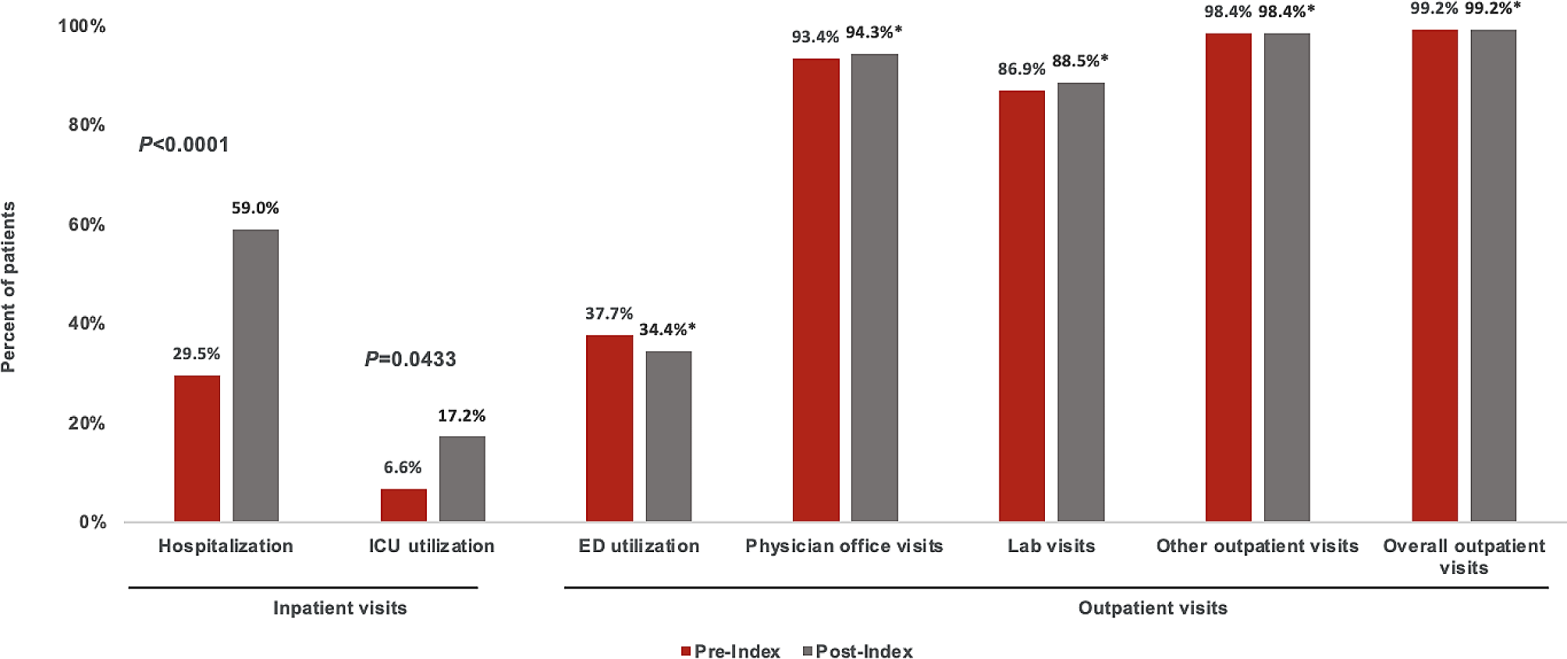
Percentage of Patients with HCRU in the 12-Month Pre- and Post-Index Period. *Not significant vs. pre-index utilization. Abbreviations: ED = emergency department; HCRU = healthcare resource utilization; ICU = intensive care unit

Almost all patients (99.2%) had any outpatient visits during the pre- and post-index periods (Fig. 4). The utilization of ED visits was 37.7% (46/122) and 34.4% (42/122); and 93.4% (114/122) and 94.3% (115/122) of patients had physician visits pre- and post-index, respectively. The mean number of overall outpatient visits was significantly higher post-index (32.9 [21.8]) relative to pre-index (24.5 [SD, 16.8]; *P* < 0.0001). This increase was driven primarily by other outpatient visits (pre-index number of visits, 12.5 [13.3]; post-index, 19.0 [17.7]; *P* < 0.0001). Finally, the mean number of unique prescriptions filled were significantly higher at 12 months post-index (46.4 [38.6]) relative to the 12-month pre-index period (37.5 [SD, 34.4]; *P* < 0.0001).

**Fig. 5:**
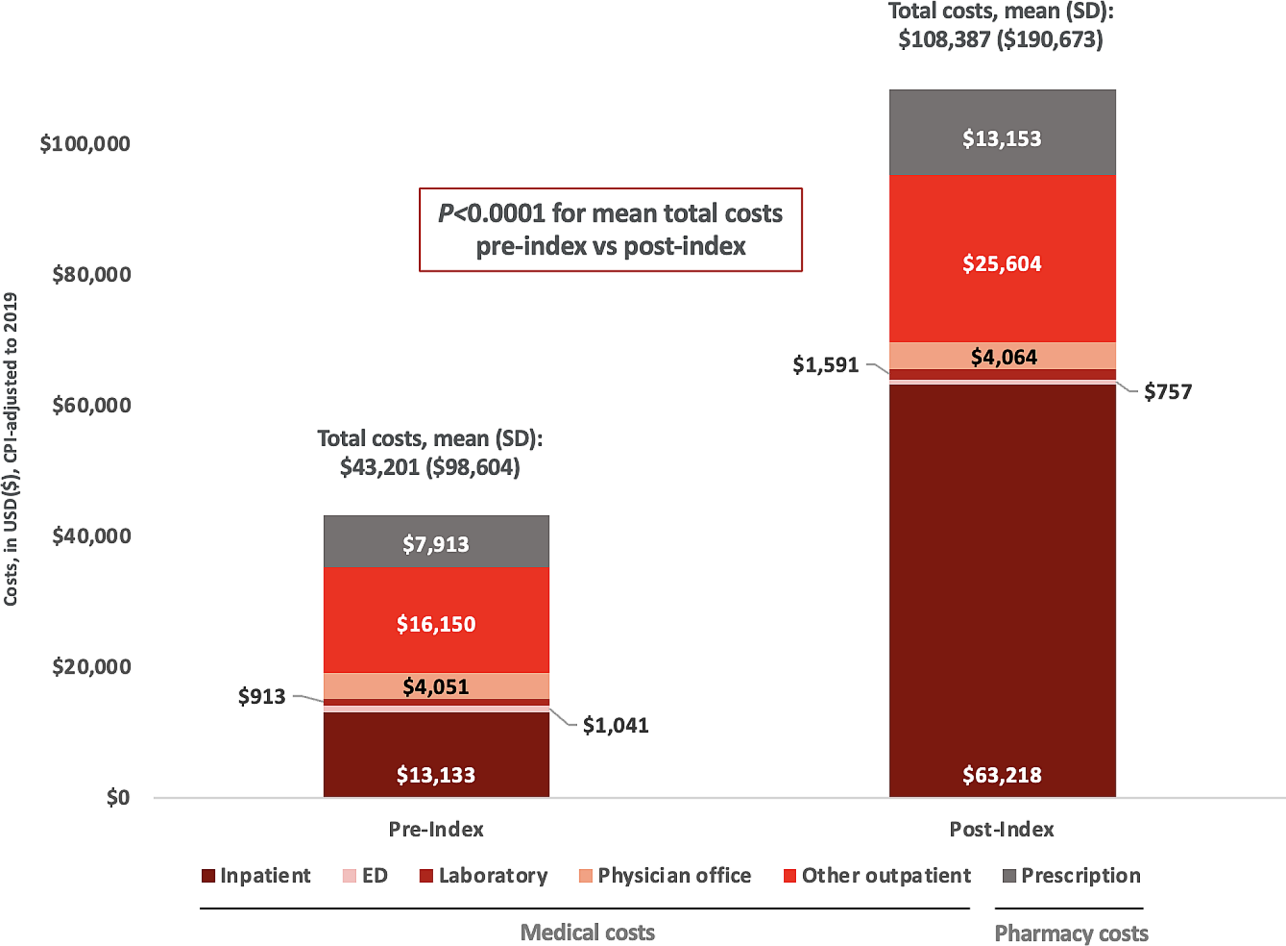
HCRU in the 12-Month Pre- and Post-Index Period, Mean All-Cause Total Healthcare Costs. Abbreviations: CPI = Consumer Price Index; ED = emergency department; HCRU = healthcare resource utilization

As shown in Fig. 5; Table 3, total healthcare costs increased significantly between the 12-month pre- and post-index periods, from a mean of $43,201 (SD, $98,604) to $108,387 ($190,673) (*P* < 0.0001). This was driven primarily by increased inpatient costs: Mean hospitalization costs were $13,133 ($28,752) pre-index, and $63,218 ($142,142) post-index (*P* < 0.0001). This included ICU costs, which were $859 ($5,497) pre-index and $7,306 ($53,436) post-index (*P* = 0.0583). There were also increased costs due to other outpatient visits (pre-index, $16,150 [$75,639], post-index $25,604 [$93,964]; *P* < 0.0001) and prescriptions ($7,913 [$15,804], $13,153 [$25,773]; *P* = 0.007).

**Table 3:** Healthcare costs in the 12-Month pre- and Post-index Period

Healthcare Resources		Pre-index (*N* = 108)*	Post-index (*N* = 108)*	*P*-value
Total healthcare costs	Mean (SD)	$43,201 ($98,604)	$108,387 ($190,673)	< 0.0001
	Median (IQR)	$20,372 ($6,476-$47,555)	$50,843 ($16,660-$100,016)	
Medical costs	Mean (SD)	$35,287 ($96,874)	$95,233 ($185,855)	< 0.0001
	Median (IQR)	$10,803 ($3,275-$37,288)	$27,166 ($12,383-$75,447)	
Inpatient costs	Mean (SD)	$13,133 ($28,752)	$63,218 ($142,142)	< 0.0001
	Median (IQR)	$0 ($0-$14,417)	$13,502 ($0-$47,646)	
ICU costs	Mean (SD)	$859 ($5,497)	$7,306 ($53,436)	0.0583
	Median (IQR)	$0 ($0-$0)	$0 ($0-$0)	
Total outpatient costs	Mean (SD)	$22,155 ($79,065)	$32,016 ($97,328)	0.0001
	Median (IQR)	$8,194 ($2,822-$17,394)	$11,695 ($6,760-$26,509)	
ED costs	Mean (SD)	$1,041 ($3,640)	$757 ($2,281)	0.7153
	Median (IQR)	$0 ($0-$680)	$0 ($0-$481)	
Laboratory costs	Mean (SD)	$913 ($1,574)	$1,591 ($5,133)	0.0894
	Median (IQR)	$329 ($61-$1,090)	$415 ($70-$1,168)	
Physician office costs	Mean (SD)	$4,051 ($9,807)	$4,064 ($7,051)	0.3897
	Median (IQR)	$1,963 ($670-$3,989)	$2,241 ($958-$4,071)	
Other outpatient costs	Mean (SD)	$16,150 ($75,639)	$25,604 ($93,964)	< 0.0001
	Median (IQR)	$3,742 ($733-$9,190)	$8,356 ($3,248-$17,256)	
Prescription costs	Mean (SD)	$7,913 ($15,804)	$13,153 ($25,773)	0.007
	Median (IQR)	$2,273 ($597-$7,061)	$4,005 ($894-$10,121)	

*Please note that 14 patients were not included in this analysis for having capitated claims.

Abbreviations: ED = emergency department; ICU = intensive care unit; IQR = interquartile range; SD = standard deviation.

## Discussion

This US medical claims analysis (2015–2019) identified real-world HCRU and costs for patients with ILD subsequently diagnosed with PH; this was accomplished by comparing patient healthcare data for the 12 months before and after PH-ILD diagnosis. Overall, both mean and median healthcare costs rose by ~ 150% from pre-index (mean, $43,201 [SD, $98,604]; median; $20,732) to post-index (mean, $108,387 [SD, $190,673]; median, $50,843) (*P* < 0.0001). This change was driven primarily by higher rates of inpatient care, more outpatient visits, and increased prescription drug use.

Post-index, inpatient care was the single greatest contributor to HCRU, and represented 58.3% of total healthcare costs. During the study, the proportion of patients requiring inpatient hospital visits increased by 100% and the proportion requiring an ICU visit increased by 161% (from 29.5 to 59.0% of patients, and 6.6–17.2%, respectively). Related to outpatient care, nearly all patients had outpatient visits both pre- and post-index (99.2%), but the mean number of outpatient visits increased significantly in the year following PH-ILD diagnosis (from 24.5 to 32.9; *P* < 0.0001). After PH-ILD diagnosis, patient prescriptions increased by 24%, corresponding to a 66% increase in mean drug costs between the pre- and post-index periods (from $7,913 to $13,153). However, a relative reduction was observed in drug prescriptions as a proportion of total healthcare costs (from 18.3 to 12.1%). These data support prior research showing high costs and healthcare burden associated with these conditions [3, 28], and suggest that the additive diagnosis of PH to ILD substantially increases existing health utilization and cost burden.

Prior to this analysis, only one US retrospective claims study existed to quantify treatment patterns and costs in patients with Group 3 PH [28]. This previous study used a case-control design to evaluate commercial and Medicare data from 2010 to 2013 using the Truven Health Database. Patients with Group 3 PH were identified based on ≥ 2 claims for PH, ≥ 1 claim for RHC or electrocardiogram, and ≥ 1 claim for a lung disease associated with Group 3 PH. Control patients had no claims for PH, but did have ≥ 1 claim for a lung disease associated with Group 3 PH. In the final study population, 19.6% of patients had PH-ILD. Over 12 months of follow-up, patients with Group 3 PH had significantly higher healthcare utilization (both overall and respiratory-related) compared to control patients, leading to all-cause healthcare costs of $44,732 for Group 3 PH and $7,051 for control patients. The primary drivers of post-diagnostic HCRU for Group 3 PH patients were inpatient admissions (35.4%), prescription drugs (33.0%), and outpatient visits (26.5%). Patients with Group 3 PH showed a steady increase in costs from baseline to follow up, while control patients with other lung diseases showed healthcare cost reductions over time.

The present analysis extends the research base for PH-ILD by providing current, targeted real-world HCRU and cost data for this patient population. Previous studies observed broader Group 3 PH which observed cohorts with different respiratory conditions aside from ILD. This study utilized a series of eligibility requirements to select a true PH-ILD population. Specifically, patients were required to have an initial ILD diagnosis, followed by a PH diagnosis, while patients with non-ILD Group 3 PH or other PH Group diagnoses were excluded.

In the current study, the most commonly prescribed drug classes, both pre- and post-index, were corticosteroids (53.3% post-index) and calcium channel blockers (36.9% post-index), and the use of antifibrotic and PAH-specific medications was rare (post-index utilization of ERA and PDE5i drugs was 1.6% and 5.7%). In the prior analysis, the most commonly prescribed drug classes post-index for patients with PH-ILD (*n* = 438) were diuretics (53.9%) and calcium channel blockers (39.3%); approximately 20% and 30% of patients received ERA and PDE5i drugs [28]. This difference observed is likely due to differing selection criteria where this study did not allow for other Group 3 associated conditions aside from ILD.

This study and its findings are subject to certain limitations associated with retrospective claims analyses. Specifically, these data were originally captured for reimbursement and not research purposes, and inaccuracies may exist in diagnostic coding or pharmacy claims. Furthermore, the data collected might be limited to insured patients captured within the database. Generalizability to patients covered outside this database and uninsured patients should be interpreted with caution. The exclusion of patients with any medical claim diagnosing a non-ILD Group 3 PH condition or a Group 2, 4, or 5 PH is a limitation, as it may have led to misclassification and therefore exclusion of some patients with PH-ILD. Similarly, the low rate of confirmatory PH diagnoses using RHC may have led to an underestimation or misclassification of patients with PH-ILD. It is possible that patients did not receive RHC because, prior to the FDA approval of inhaled treprostinil for PH-ILD, no change in treatment would have been likely, even with a definitive diagnosis. Regardless, the low rate of RHC at index points to a limitation in current clinical practice and a knowledge gap regarding the approaches used by clinicians to confirm, and subsequently file claims, for PH.

In conclusion, regardless of its etiology, PH is a costly and complex disease. However, patients with ILD appear to experience a uniquely high medical and healthcare cost burden after diagnosis of pulmonary hypertension. The timely management and treatment of PH-ILD is needed to mitigate the clinical and economic consequences of disease progression.

## Data Availability

The data that support the findings of this study are available from Merative (Ann Arbor, MI, USA) but restrictions apply to the availability of these data, which were used under license for the current study, and so are not publicly available. Data are however available from the authors (Benjamin Wu, PharmD, MS) upon reasonable request and with permission of Merative.

## Abbreviations

ED: emergency department
ICU: intensive care unit
IQR: interquartile range
SD: standard deviation

## List of abbreviations

6MWD: 6-minute walk distance
CCAE: Commercial Claims and Encounters Database
CPT: Current Procedural Terminology
ED: emergency department
ERA: endothelin receptor antagonists
FDA: United States Food and Drug Administration
HCRU: healthcare resource utilization
ICU: intensive care unit
ILD: interstitial lung disease
IPF: idiopathic pulmonary fibrosis
IQR: interquartile range
LOS: length of stay
mPAP: mean pulmonary artery pressure
PAH: pulmonary arterial hypertension
PDE5i: phosphodiesterase-5 inhibitor
PH: pulmonary hypertension
Quan-CCI: Quan-Charlson comorbidity index
RHC: right heart catheterization
SD: standard deviation
sGCS: soluble guanylate cyclase stimulator

## References

[1] King CS, Nathan SD (2019). Pulmonary hypertension due to interstitial lung disease. Curr Opin Pulm Med.

[2] Simonneau G, Montani D, Celermajer DS, Denton CP, Gatzoulis MA, Krowka M, et al. Haemodynamic definitions and updated clinical classification of pulmonary hypertension. Eur Respir J. 2019;53(1). 10.1183/13993003.01913-2018. 10.1183/13993003.01913-2018 PMC6351336 30545968

[3] Wijsenbeek M, Suzuki A, Maher TM. Interstitial lung diseases. Lancet. 2022;400(10354): 769 – 86. 10.1016/S0140-6736(22)01052-2. 10.1016/S0140-6736(22)01052-2 35964592

[4] Nathan SD, Shlobin OA, Ahmad S, Koch J, Barnett SD, Ad N (2008). Serial development of pulmonary hypertension in patients with idiopathic pulmonary fibrosis. Respiration.

[5] Dhont S, Zwaenepoel B, Vandecasteele E, Brusselle G, De Pauw M. Pulmonary hypertension in interstitial lung disease: an area of unmet clinical need. ERJ Open Res. 2022;8(4). 10.1183/23120541.00272-2022. 10.1183/23120541.00272-2022 PMC9661248 36382238

[6] Lederer DJ, Arcasoy SM, Wilt JS, D’Ovidio F, Sonett JR, Kawut SM (2006). Six-minute-walk distance predicts waiting list survival in idiopathic pulmonary fibrosis. Am J Respir Crit Care Med.

[7] Nathan SD, Shlobin OA, Ahmad S, Urbanek S, Barnett SD (2007). Pulmonary hypertension and pulmonary function testing in idiopathic pulmonary fibrosis. Chest.

[8] Shorr AF, Wainright JL, Cors CS, Lettieri CJ, Nathan SD (2007). Pulmonary hypertension in patients with pulmonary fibrosis awaiting lung transplant. Eur Respir J.

[9] Minai OA, Santacruz JF, Alster JM, Budev MM, McCarthy K (2012). Impact of pulmonary hemodynamics on 6-min walk test in idiopathic pulmonary fibrosis. Respir Med.

[10] Rivera-Lebron BN, Forfia PR, Kreider M, Lee JC, Holmes JH, Kawut SM (2013). Echocardiographic and hemodynamic predictors of mortality in idiopathic pulmonary fibrosis. Chest.

[11] Kimura M, Taniguchi H, Kondoh Y, Kimura T, Kataoka K, Nishiyama O (2013). Pulmonary hypertension as a prognostic indicator at the initial evaluation in idiopathic pulmonary fibrosis. Respiration.

[12] Raghu G, Amatto VC, Behr J, Stowasser S (2015). Comorbidities in idiopathic pulmonary fibrosis patients: a systematic literature review. Eur Respir J.

[13] Hamada K, Nagai S, Tanaka S, Handa T, Shigematsu M, Nagao T (2007). Significance of pulmonary arterial pressure and diffusion capacity of the lung as prognosticator in patients with idiopathic pulmonary fibrosis. Chest.

[14] Morrow LE, Hilleman D, Malesker MA (2022). Management of patients with fibrosing interstitial lung diseases. Am J Health Syst Pharm.

[15] King TE, Behr J, Brown KK, du Bois RM, Lancaster L, de Andrade JA (2008). BUILD-1: a randomized placebo-controlled trial of bosentan in idiopathic pulmonary fibrosis. Am J Respir Crit Care Med.

[16] King TE, Brown KK, Raghu G, du Bois RM, Lynch DA, Martinez F (2011). BUILD-3: a randomized, controlled trial of bosentan in idiopathic pulmonary fibrosis. Am J Respir Crit Care Med.

[17] Raghu G, Behr J, Brown KK, Egan JJ, Kawut SM, Flaherty KR (2013). Treatment of idiopathic pulmonary fibrosis with ambrisentan: a parallel, randomized trial. Ann Intern Med.

[18] Raghu G, Million-Rousseau R, Morganti A, Perchenet L, Behr J, Group MS (2013). Macitentan for the treatment of idiopathic pulmonary fibrosis: the randomised controlled MUSIC trial. Eur Respir J.

[19] Corte TJ, Keir GJ, Dimopoulos K, Howard L, Corris PA, Parfitt L (2014). Bosentan in pulmonary hypertension associated with fibrotic idiopathic interstitial pneumonia. Am J Respir Crit Care Med.

[20] Seibold JR, Denton CP, Furst DE, Guillevin L, Rubin LJ, Wells A (2010). Randomized, prospective, placebo-controlled trial of bosentan in interstitial lung disease secondary to systemic sclerosis. Arthritis Rheum.

[21] Zisman DA, Schwarz M, Anstrom KJ, Collard HR, Flaherty KR, Idiopathic Pulmonary Fibrosis Clinical Research Network (2010). A controlled trial of sildenafil in advanced idiopathic pulmonary fibrosis. N Engl J Med.

[22] Jackson RM, Glassberg MK, Ramos CF, Bejarano PA, Butrous G, Gomez-Marin O (2010). Sildenafil therapy and exercise tolerance in idiopathic pulmonary fibrosis. Lung.

[23] Nathan SD, Behr J, Collard HR, Cottin V, Hoeper MM, Martinez FJ et al. RISE-IIP: Riociguat for the treatment of pulmonary hypertension associated with idiopathic interstitial pneumonia. Eur Respir J. 2017;50: OA1985. 10.1183/1393003.CONGRESS-2017.OA1985.

[24] TYVASO DPI. Prescribing information, United Therapeutics Corp. 2022. Accessed 16 March 2023. https://www.tyvaso.com/pdf/TYVASO-DPI-PI.pdf.

[25] Wexler M, Tyvaso. April DPI approved by the FDA for PAH and PH-ILD. https://pulmonaryhypertensionnews.com/news/tyvaso-dpi-approved-fda-pah-ph-ild/. Accessed 21 2023.

[26] Waxman A, Restrepo-Jaramillo R, Thenappan T, Ravichandran A, Engel P, Bajwa A (2021). Inhaled treprostinil in pulmonary hypertension due to interstitial lung disease. N Engl J Med.

[27] Olson AL, Hartmann N, Patnaik P, Garry EM, Bohn RL, Singer D (2022). Healthcare resource utilization and related costs in chronic fibrosing interstitial lung diseases with a progressive phenotype: a US claims database analysis. Adv Ther.

[28] Heresi GA, Platt DM, Wang W, Divers CH, Joish VN, Teal SA (2017). Healthcare burden of pulmonary hypertension owing to lung disease and/or hypoxia. BMC Pulm Med.

[29] Heresi GA, Dean BB, Castillo H, Lee HF, Classi P, Stafkey-Mailey D (2022). Identifying patients with Group 3 pulmonary hypertension associated with COPD or ILD using an administrative claims database. Lung.

[30] Quan H, Li B, Couris CM, Fushimi K, Graham P, Hider P (2011). Updating and validating the Charlson comorbidity index and score for risk adjustment in hospital discharge abstracts using data from 6 countries. Am J Epidemiol.

[31] Centers for Medicare & Medicaid Services (CMS). Capitation and pre-payment. https://innovation.cms.gov/key-concept/capitation-and-pre-payment. Accessed 21 April 2023.

[32] U.S. Bureau of Labor Statistics. Consumer Price Index. https://www.bls.gov/cpi/factsheets/medical-care.htm. Accessed 21 April 2023.

